# Cultivating climate resilience in California agriculture: Adaptations to an increasingly volatile water future

**DOI:** 10.1073/pnas.2310079121

**Published:** 2024-07-29

**Authors:** Josué Medellín-Azuara, Alvar Escriva-Bou, Amélie C. M. Gaudin, Kurt A. Schwabe, Daniel A. Sumner

**Affiliations:** ^a^Civil and Environmental Engineering, University of California, Merced, CA95343; ^b^Civil and Environmental Engineering, University of California, Los Angeles, CA90095; ^c^Department of Plant Sciences, University of California, Davis, CA95616; ^d^School of Public Policy, University of California, Riverside, CA92501; ^e^Agricultural and Resource Economics, University of California, Davis, CA95616

**Keywords:** climate change, agriculture, sustainability, California, groundwater

## Abstract

California agriculture will undergo significant transformations over the next few decades in response to climate extremes, environmental regulation and policy encouraging environmental justice, and economic pressures that have long driven agricultural changes. With several local climates suited to a variety of crops, periodically abundant nearby precipitation, and public investments that facilitated abundant low-priced irrigation water, California hosts one of the most diverse and productive agroecosystems in the world. California farms supply nearly half of the high-nutrient fruit, tree nut, and vegetable production in the United States. Climate change impacts on productivity and profitability of California agriculture are increasing and forebode problems for standard agricultural practices, especially water use norms. We highlight many challenges California agriculture confronts under climate change through the direct and indirect impacts on the biophysical conditions and ecosystem services that drive adaptations in farm practices and water accessibility and availability. In the face of clear conflicts among competing interests, we consider ongoing and potential sustainable and equitable solutions, with particular attention to how technology and policy can facilitate progress.

## The Evolution of California Agriculture and Climate

### California Agriculture: A Brief History.

California’s Mediterranean climate, albeit highly variable with frequent periods of drought and floods, provided the foundation for a diverse and vibrant agricultural industry to grow in response to the availability of low-cost labor and water supplies. Starting in the middle of the 19th century field crops–grains, forages, and cotton–dominated California crop landscapes, if not value of production, for a hundred years. Toward the beginning of the 20th century, though, California agriculture began its move toward intensive cropping of vegetables and fruits. Railroads helped expand produce markets and low-wage immigrant labor. Later, migrants from the Dust Bowl, and then from Mexico, kept labor costs on fruit and vegetable farms competitive (1).

Importantly, irrigation infrastructure and regulation—particularly water pumping, storage, transport, and rules of use—allowed cultivation of water-intensive summer crops where no rain fell for 6 mo each year. The Great Depression catalyzed massive surface water infrastructure developments such as the Colorado River Project and, in the late 1930s, the Central Valley Project (CVP) by the US Bureau of Reclamation (2). Further growth of infrastructure in the postwar era included the State Water Project (SWP) serving mostly cities and some agricultural lands in central and southern California. Infrastructure development over this period created one of the largest and most engineered irrigated agricultural systems in the world. The water supply network bridged the gap of hundreds of kilometers between the water-rich north—with mountains and heavy precipitation in the winter—and the low-precipitation Mediterranean climate central and south that plays host to most of California agriculture production and population.

For nearly two centuries California farms have prospered through technological adoption, innovation investments, and on-farm management improvements. Yet with a changing climate coupled with increased concerns over the environment and sustainability, the landscape of California agriculture is changing. Over the past two decades, noticeably less land and water has been devoted to extensive field crops, as farms shifted to vegetables and tree and vine crops. These specialty crops generally produce higher revenues per unit of land and water (1). Expectations of higher returns have contributed to more than half of the state’s irrigated agricultural croplands growing fruits, nuts, and vegetables, which comprise roughly 80% of the farm revenue and employment (3). The degree to which these changes and concerns significantly reduce agriculture’s presence and productivity will depend on how Californians, including its growers and policymakers, respond.

### The Geography and Character of California Agriculture.

The mosaic of agriculture in California is driven by a variety of natural and human-created conditions (*SI Appendix*, Fig. S1 ). California’s terrain, climate, and soil heterogeneity are instrumental to California’s diverse array of agricultural commodities. The irrigated crop footprint alone is nearly 3.8 million hectares (ha). Land in farms spans more than 10 million ha, producing over 400 crop and livestock commodities that annually generate around $50 billion in cash receipts and support 420,000 jobs in 2021 (4). The food and beverage processing sector, which primarily relies on local crop and animal supplies, supports an additional 250,000 jobs. Agriculture contributes significant shares of the income and employment in areas such as the CV, where labor, capital, irrigation water, management, and downstream sectors in livestock and food processing are closely linked.

We briefly describe agriculture in three regions comprising the largest areas of irrigated acreage and commodity value: the CV, the Southern California Region, and the Coastal California. Agriculture in California’s foothills and mountain areas provide nearly 5 million ha of pasture and hay for cattle, along with the winter snowpack that historically stores nearly a third of California’s runoff that supplies CV irrigation as well as urban water use.

#### CV.

The nearly 52,000 km^2^ CV accounts for more than two-thirds of California’s irrigated agriculture, encompassing a few major cities and dozens of moderate-sized rural communities. The northern part of the CV contains the Sacramento Valley and the Sacramento River basin, which averages 890 mm/y. of precipitation and is close to the snowpack-heavy northern mountains. The Sacramento Valley grows rice, tree nuts and fruits, tomatoes, alfalfa, and dozens of other crops. This region applies around 10,100 hm^3^ of water for irrigation of over 839,000 ha.

The southern and larger part of the CV, the San Joaquin Valley (SJV), includes the San Joaquin River Basin with low average precipitation (310 mm/y), and the Tulare Lake Basin with slightly more (373 mm/y) precipitation. The SJV has even more crop diversity than the north. It has experienced steady declines in field crops such as cotton, grains, and alfalfa that have been replaced by tree nuts. The SJV applies around 22,860 hm^3^ of water for irrigation of over 2 million ha. The west side of the southern SJV is affected by soil salinization due to the rising water table above the Corcoran clay layer and poor drainage (5, 6). Water supplies for irrigation come from the Sierra Nevada and northern basins runoff delivered through local, state, and federal water projects as surface water and from local groundwater. The Sacramento River and the San Joaquin River basins drain to the Sacramento San Joaquin Delta (SSJD), which also serves as the water supply hub agricultural and urban use in the SJV and some coastal areas.

#### Coastal California.

The south and central parts of the long coastal region, about 1,200 km from San Diego to Oregon, contain a series of relatively cool and agriculturally intensive valleys producing high revenue-per-ha crops such as citrus, avocados, berries, fresh vegetables, greenhouse and nursery crops, and high-priced wine grapes. Coastal irrigation is mostly supplied by pumping from coastal aquifers and small surface water diversion projects. Annual precipitation in the Coastal hydrologic regions varies widely between 250 and 2,500 mm/y in the north coast, but with much lower average annual precipitation in the south coast (340 mm/y) (7). Similar to precipitation, mean annual temperatures are much more varied along the coast relative to the CV. The coastal counties account for about 25% of total farm revenue in California and apply roughly 3,500 hm^3^ of irrigation water over 543,000 ha.

#### Southern California.

The Southern California region consists of a significant and broad array of cropping systems and water sources and includes the counties of Los Angeles, Riverside, San Bernardino, San Diego, Orange, and Imperial. It overlaps with the southern portions of Coastal California. Nowhere in this region are the conflicts and possible consequences of climate change on irrigated agriculture more challenging than in the Imperial Valley, which borders Mexico to the south, Arizona to the east, and San Diego County to the west. While the region receives less than 75 mm/y of precipitation on average, senior water rights to the Colorado River coupled with an extended growing season and warm climate enable a diverse range of crops (8).

Within Imperial County sit two of the most senior water rights holders for Colorado River water—the Imperial Irrigation District (IID) and the Palo Verde Irrigation District (PVID). Of the 5,400 hm^3^ California annual allocation Colorado River Compact, IID’s claim is 3,800 hm^3^ while PVID’s allocation is roughly 420 hm^3^ annually in applied water. The region also hosts the Salton Sea, the largest lake in California, a terminal hypersaline lake receding due to lower inflows.

## Challenges Faced by California Agriculture under a Changing Climate

Climate factors and adaptation determine the viability of California agriculture. First, warming influences the form of precipitation (rain or snow), and the rate and seasonality of mountain snowmelt both of which affect the timing and intensity of runoff. Warming also constrains water supplies, increases water demands, and affects other biophysical components of crops. Third, climate change is also aggravating some of the entrenched conflicts between California’s agriculture and other sectors. And fourth, the climate vulnerability of California’s agriculture is also partly determined by evolving global market demand which is also affected by climate change.

### Effects of Climate-Related Stressors.

Increase in temperatures and alteration of precipitation patterns, including the reduction of precipitation falling as snow, are having direct impacts on California agriculture. Sea-level rise and atmospheric carbon concentration may also affect water supplies and growing conditions and yields for some crops and regions (9).

#### Increased evaporative demands.

Higher temperatures and atmospheric moisture deficit may increase soil evaporation and crop irrigation requirements. This furthers the gap between water availability and demand contributing to increased scarcity. In California, reference evapotranspiration (ET_o_) increased between 50 and 100 mm/y during the 1980 to 2020 period, with higher temperatures contributing more than 70% of the rise (10). During the 2022 drought, similar increases in annual ET_o_ were estimated across the state due to antecedent dry soil conditions and a thirsty atmosphere (11). Higher crop water demands are expected to continue while warming persists, increasing the likelihood of evapotranspiration-induced droughts (12).

#### Change in water availability.

While California climate projections do not show a clear trend in average annual precipitation (13), the alteration of precipitation patterns—including precipitation volatility and rain/snow patterns—are affecting water supplies, particularly the seasonal availability. Precipitation volatility is causing more intense swings between dry and wet periods. Anthropogenic forcing is found to yield large twenty-first-century increases in the frequency of wet extremes, and smaller but statistically robust increases in dry extremes (14). Given California’s reliance on large reservoir storage, this could result in less water available during the irrigation season as reservoirs would likely release more water during winter for flood protection.

Recent studies highlight that the seasonal snowpack is receding considerably and will likely continue receding with warming (14). The Sierra Nevada mountains function as a natural reservoir: They store water during winter and spring when reservoirs need to be partly empty for flood protection, and supply irrigation during the spring and summer, especially for the CV crops. By 2050, snowpack in the Sierra Nevada’s–which historically provides approximately 30% of California’s annual supply, is expected to decline by as much as 45% (15). With less snow and earlier melting, the function of the snowpack must change (16), and adaptation measures in the form of reservoir reoperation may reduce supply losses (17).

#### Sea level rise.

Rising sea levels are constraining water supplies in at least two ways. First, sea level rise may increase salinity in some areas of the SSJD, a hub from which California’s two major water projects (SWP and the CVP) pull supplies, compromising water deliveries to the south and creating water quality–driven deficits in supply for agriculture and cities (18). Second, in coastal areas that rely on groundwater for irrigation, sea level rise increases the risk of saline intrusion. This has already affected aquifers in the central and south coasts of California (19).

#### Other climate effects on California’s crops.

A complex crop pattern in California presents a challenge to predict future climate impacts on crop yields. Research suggests a potentially broad decline in the productivity of some specialty crops, including vines, nuts, and citrus (9). With the exception of alfalfa, declines in the yields of field crops will likely occur as well, particularly toward the end of the twenty-first century. Impacts of warmer climate, including fewer winter chill hours, may affect yields in tree crops (9). Conversely, some studies argue that higher atmospheric carbon concentrations may lead to increased yields in some crop varieties (16).

Projected declines in crop yields have also been linked to new or more abundant pests, diseases, and invasive species in response to climate-related shifts in the resources, habitat, and ecological interactions that regulate their distribution, abundance, and behaviors (9, 20). Increase in pest pressure, altered disease dynamics, and greater vulnerability of drought-stressed plants to pest and disease infections will further stress cropping systems, especially since overreliance on common biocides may result in ineffective pest control. Periodic crop failures or generally lower yields of specialty crops may also occur as rising temperatures and water limitations inhibit pollinator activity and synchrony of plant and pollinator life cycles (21). These impacts place additional importance on research and development to help California, and agricultural globally, adapt effectively to climate change through innovation.

### California Agriculture Conflicts with Other Sectors.

Conflicts over water use are a global phenomenon, with recorded incidences dating back to 5000 BC (22). With climate change resulting in an increase in water scarcity such conflicts are likely to be exacerbated (23). Limiting conflict requires significant changes in governance and cooperation, which has been difficult to achieve. California’s water conflicts were immortalized in the 1974 movie, “Chinatown,” which provided a fictionalized account of the conflict involving the Los Angeles Department of Water and Power and Owens Valley ranchers in the early 20th century (24). Agriculture, municipal (urban) users and environmental demands have been in constant competition for increasingly scarce water. This is no surprise given that 40% of all water in California—and 80% of consumed water—is used by irrigated agriculture (42 hm^3^/y), percentages broadly consistent with irrigation use in other arid and semiarid regions globally (25). Conflict in two California regions stands out given the size of the agricultural industry and its historic water rights, and how water use and rights are at odds with environmental flows, municipal growth, and a better understanding of sustainable water availability.

#### Central Valley.

##### Agriculture and environment.

Reduced water in streams due to increased diversions and degraded water quality from nonpoint source pollution have compromised water supplies for ecosystems and communities in California. In the late 1960s, operation of the CVP and SWP (with the main hub in the SSJD) made evident a sharp decline in native fish populations attributed to lower and quality-degraded streamflow (26). In response, regulations were developed requiring state and federal agencies to modify operations and set water quality standards to protect fish and wildlife, which decades later resulted in the CVP Improvement Act (CVPIA) of 1992 (27). The tension between water quality protection and water allocation for users statewide, including in-Delta farming, persisted as higher SSJD outflows were accomplished by reducing SSJD upstream diversions and/or reducing SSJD exports south. These environmental conflicts with agricultural production, particularly over water use, will likely increase under climate change as drought intensity and frequency increase.

##### Conflicts among agricultural-related sectors.

As in other regions, periodic cuts in surface water access have been partially offset by increased groundwater pumping. In California, such drought-related increases in pumping added to the existing CV overdraft of roughly 2,000 hm^3^ per year. An increasing number of domestic wells have gone dry given rural communities well depths typically are not as deep as agricultural operators (28), disproportionately affecting disadvantaged rural communities, particularly in the SJV. In addition, degraded groundwater water quality due to nitrate contamination from fertilizer overapplication has further compromised safe drinking water supplies in many rural locations lacking economies of scale for adequate water treatment (29).

The Sustainable Groundwater Management Act (SGMA) of 2014 mandated long-term reductions in consumptive groundwater use through locally developed groundwater sustainability plans. When coupled with the effects of environmental regulations and climate change, meeting these long-term reductions will necessarily mean less irrigated land with estimates ranging between 180 and 350 thousand fewer hectares (30).

While SGMA does not require groundwater users meet sustainability plans until early 2040s, water restrictions over the last decade since SGMA passage highlight the challenges that will confront irrigated agricultural operators in the SJV. Continued irrigation of crops such as fruits, tree nuts, and vegetables during recent droughts has come at the expense of increased fallowing of field crops. Higher short-term net revenues per unit of water and the threat of significant loss in capital from not irrigating trees and vines underlie the economics of such decisions when water cuts are expected to be temporary. Despite these pressures, water-intensive forage crops, such as alfalfa hay and corn silage, continue in the SJV to feed the 90% of California’s dairy cows that reside there. Because of high transport cost per unit of value, much of the alfalfa hay and all the corn silage is grown near dairy farms even in areas with little to no access to surface water. These economic pressures from increased area of tree nut orchards and dairy forage demand have made meeting local groundwater sustainability in parts of the SJV especially challenging.

#### Southern California.

##### Agricultural and urban interactions.

Colorado River water users are negotiating new water-sharing agreements that are intended to govern Colorado River operations and management beginning in 2026 and will likely result in reductions of lower Basin state allocations, including California’s, to bring the Basin back into long-term balance under climate change (31). How those reductions are allocated among Imperial Valley agriculture—the single largest user of Colorado River water—and the two largest southern California wholesale water agencies—Metropolitan Water District of Southern California (MWD), supplying 19 million people, and the San Diego County Water Authority (SDCWA), supplying 3.3 million people, will create challenges.

While the reductions will create conflict, as evidenced by past lawsuits among these agencies, opportunities will also arise, including building upon past agricultural–urban water transfers. For example, in the early 1990s IID and MWD agreed to a long-term transfer of roughly 12,950 hm^3^ of water annually. In 2003, and in response to the USBR requiring California reduce its use of surplus Colorado River water due to demand growth in Arizona and Nevada, the largest agricultural-municipal water transfer in California was signed as part of the Quantification Settlement Agreement (QSA) (32). Under the QSA, IID agreed to transfer up to 25,000 hm^3^ of water annually—generated through water conservation—to SDCWA, which due to lower priority water rights to the Colorado was to lose its allocation with the elimination of California’s surplus usage.

##### Agriculture and environment interactions.

A constant challenge confronting agricultural water transfers involving IID is the impact of those transfers on the Salton Sea. As a highly saline terminal lake dependent on IID irrigation runoff (which provide 85% of the inflows), its volume and surface area will fluctuate with changes in applied irrigation in IID. Consequently, as water transfers from IID to MWD or SDCWA increase, or if water allocations to IID decrease, the Salton Sea will shrink and more playa will be exposed with significant negative externalities on the environment and local communities surrounding the Salton Sea (32). Such concerns were a primary reason IID withheld its support for the Drought Contingency Plan of 2019 involving California, Arizona, Nevada, and the USBR and, subsequently, sued MWD—under the California Environmental Quality Act—for signing the agreement and aimed to cover California’s share of the 2019 Plan reductions. While the lawsuit was dropped 2 y later, is emblematic of the environmental challenges irrigated agriculture will confront more regularly under climate change.

### California Agriculture within Global Markets and Institutions.

Adaptation of California agriculture to climate change will proceed within the broader context of global climate change and global impacts on input and farm product markets. Climate change outside California influences the future of California agriculture by affecting economic prospects and hence choices of its farms. Any assessments of direct climate impacts on California productivity and water availability across farm commodities must consider these same impacts on agriculture supply conditions elsewhere, and hence the global market conditions for California grown commodities. Crops that face more intense competition in global markets (e.g., walnuts compared to pistachios) may face more losses from increased production costs under climate change unless there are even larger negative climate impacts in competitive regions. Assessment of climate change–related impacts on the evolution of California agriculture will be inherently affected by demand and supply conditions in local and global markets that are affected by climate changes elsewhere.

Furthermore, impediments to global market access influence the functioning of markets and hence the impacts of climate change on California agriculture. Such impediments can positively or negatively affect agricultural production in California through their impacts on the demand for California exports (33). For example, Iran was the major competitor for California pistachios but lost market share in recent decades, in part due to sanctions and trade barriers, which created opportunities for California pistachios.

Agricultural subsidies and trade measures favor certain commodities, farm practices, and growing regions, relative to others. U.S. farm subsidies (including of crop insurance subsidies) tend to be low relative to global standards (34) and have declined steadily until big jumps from ad hoc subsidies from 2019 through 2021. Because U.S. subsidies for the vegetables, fruits, and tree nuts grown in California tend to be relatively low, farm subsidies have had modest direct effects on production patterns in California in recent years, yet subsidies and trade barriers elsewhere do affect export market opportunities that are shifting with climate change.

Climate change–related impacts affect costs and returns to crop insurance programs, which are controlled and subsidized by the USDA (35, 36). Crop insurance is almost always highly subsidized. Farms pay less than half the costs of U.S. crop insurance. Farms generally enroll only if their insurance premium payments are far below expected farm payouts. Highly subsidized federal crop insurance programs may reduce incentives for adoption of climate-resilient farm practices, particularly if insurance payoffs exceed climate impacts on crop yields. By shifting climate-related costs away from farms, crop insurance subsidies may delay farm adaptations (35). At the same time, as climate change raises yield or price variability it may increase crop insurance subsidies (36). Nonetheless, even high crop insurance subsidy rates comprise a small fraction of farm revenue and thus have only modest effects on cropping patterns and, consequently, likely only small effects on adaptation to climate change.

Other farm subsidies and trade measures increasingly recognize climate change adaptation and GHG mitigation (37). The EU has begun to regulate farms to reduce GHG emission and propose farm import tariffs to impose parallel costs on imports. However, such policies may mask traditional protectionist barriers that could exacerbate losses from climate change (37). Finally, California agriculture could gain from foreign adoption of carbon taxes and other measures because it often has a lower carbon footprint than its competitors due to relatively high productivity per unit of output.

## Promising Mitigation and Adaptation Strategies: The Role of Science, Technology, Institutions, and Cooperation

California agriculture likely confronts a future defined by higher temperatures and both lower and less certain water supplies for irrigation. As such, the ability of California agriculture to thrive in the future will depend on its ability to develop mitigation and adaptation strategies to reduce vulnerabilities and increase resilience under this new climate and water regime. Farmers and other water users along with policy makers naturally consider a variety of approaches and possibilities to either reduce water scarcity itself or reduce the costs associated with it. These approaches can be categorized into three major groups: demand-side, supply-side, and institutional.

### Demand-Side Practices and Policies.

Irrigated water demand reductions can ameliorate impacts of limited water access. In deciding whether to reduce their water use, users naturally consider costs of the competing options and potential gains from such reduced use. Over the past four decades, agricultural water use in California has decreased by nearly 15% while overall farm revenue has increased by nearly 40% (25). Reduction of applied irrigation water may be largely attributed to a combination of three factors—changes in irrigation practices, changes in crop mix, and irrigated land fallowing.

#### Irrigation efficiency and scheduling.

Changes in irrigation practices usually fall into two categories: a) increases in irrigation efficiency involving a higher ratio of irrigation-fulfilled crop evaporative demand to total applied water in the current season, and b) changes in irrigation timing and quantities (or deficit irrigation) so that a higher proportion of the applied water fulfills evaporative demand. Since the 1980s, the irrigated area in California using gravity-fed irrigation (e.g., furrow or flood irrigation) has decreased by about 25% from nearly 2.5 million ha down to approximately 1.9 million ha (38). Concurrently, the amount of acreage using sprinkler irrigation has slightly declined over that period while the amount of acreage with installed drip irrigation has increased from around 121,400 ha to nearly 1.2 million ha. Augmenting supply through recovery of conveyance losses may also contribute to increasing efficiency yet will reduce deep percolation. Much of this change has accompanied a change in the crop mix from annual crops to trees and vines.

As pointed out increasingly (39), such forms of higher irrigation efficiency do not change crop evaporative requirements, but rather changes the amount of water applied by reducing the amount of return flows either as runoff, deep percolation, or both (40). Such reduced flows can have a variety of negative impacts, including i) if those deep percolation flows would otherwise recharge aquifers, ii) if the runoff had contributed to return flows for downstream users, and iii) if the runoff/deep percolation flows contributed to environmental flows and ecosystems services. In these cases, applied water reductions may not increase what might be termed “system efficiency,” and/or may result in environmental damages. Without attention to the entire water balance, government programs intended to save water via increased irrigation efficiency, as defined around applied water or diversions, rarely save water on a system-wide basis. Often apparent water savings increase water use elsewhere in the system (41, 42).

Improvements in irrigation timing to match soil water depletions may lead to significant reductions in nonbeneficial water losses without any appreciable change in crop yield, or substantial infrastructure or production cost increases (43). This strategy, which includes deficit irrigation, has seen a surge of research since the early 2000s (44) and has found no substantial crop yield changes when appropriate phenological stage-scheduling and water yield response is considered (45).

Significant systemwide potential water savings are limited with efficiency and timing strategies. Understanding whether and to what extent reductions in net water use have occurred under these strategies requires improved water accounting transparency and adoption of technologies—e.g., remote sensing—that can track water use patterns spatially and temporally. Water accounting that distinguishes between water withdrawals, consumptive use, and return flows are crucial (39).

#### Changes in the crop mix.

Over the past two decades, California has seen a significant increase in perennial crop area and a decrease in field crop area in response to expectations about long-term crop profitability and related factors (46). Crop net water requirements vary widely across California’s highly diversified agriculture with field crops (excluding pasture and alfalfa) using 58 cm/y consumptively (as evapotranspiration of applied water), vegetables 45 cm/y, trees 66 cm/y, and alfalfa and pasture 94 cm/y on average. With typically lower net returns per unit of land and water relative to tree and vegetable crops, reduced field crop area, including alfalfa and pasture, provides a lower net cost means (and thus reduced economic impact) to respond to reduced water allocations. Even higher water savings could be achieved through a switch from irrigated crops to unirrigated winter cereal crop productions, as shown in ref. 47. These crops–or other traditionally rainfed crops that could be supported by additional marginal irrigation–may present an opportunity for maintaining agricultural lands while reducing the agricultural water footprint. Such evaluation would need to also consider the lower net returns of rainfed crops.

While water use reductions may occur through changes in crop mix, we caution against a heavy regulatory approach in determining what crops are grown. Reductions in the availability of water would create incentives for growers to change their crop mix based on their business calculus. Pricing water at a rate that more accurately represents its scarcity value (rather than based solely on the costs of delivering the water) would also provide incentives for growers to shift their operations to less water-intensive crops (48, 49). Current laws and regulation often require water prices to represent water delivery costs alone, which constitute a small fraction of crop production costs. As a result, only large increases in water prices from current rates would provide sufficient incentive for significant changes in crops.

#### Land idling and repurposing.

Reduced irrigation to address groundwater depletion and ongoing and future climate change impacts will likely lead to significant declines in irrigated cropland planting, with some repurposing to nonagricultural uses. In the SJV, average annual irrigation water supplies are likely to decline by 20% by 2040 from 2010 levels. Simulations indicate this would trigger a reduction in 180 to 350 thousand hectares (10 to 19%) of irrigated cropland–depending on the quantity of new water supplies that could be brought to the SJV (30).

Reducing agricultural irrigated areas substantially comes with consequences that are not fully understood. First, to what extent will the local economy decline, and what options can mitigate such effects. Reductions in local farm and nonfarm employment and income are a real concern without timely and effective transitions to other job-creating production (11). Second, such land use changes likely create downstream impacts on food prices and regional/global markets. California produces a significant share of U.S. consumption of many fruit, vegetable, and tree nut crops, but these high-revenue per irrigated acre crops are least likely to face cuts from water scarcity. Price impacts also depend on global competition and climate effects in other supply areas. Historically, specialty crop production in California has been relatively stable during droughts, and thus such price effects have been minimal (11). The nationally important SJV dairy supplies, which rely on locally irrigated forage crops, may also cause national price impacts.

A widely cited case of the third-party effects of reduced water use through idling and transfers is the Colorado Big Thompson (50). Factors such as the degree of diversification in a region’s economy, prosperity in the region, as well as the size, number, and conditions of the transfers play a role in influencing the magnitude of the regional impacts. Concerns over third-party effects were instrument in IID’s decision to put conditions on its water transfers under the QSA—they limited the extent of land fallowing and required that water transfers to eventually be sourced from on-farm conservation. Strategies to combat such concerns over third-party effects likely involve a variety of approaches including social programs and support for land repurposing (50).

Land repurposing as a response to the likely reductions in irrigated cropland is gaining significant attention in California (51). Developing solar energy, restoring desert and upland habitat, or riparian and wetland areas, expanding water-limited crops, or developing water-efficient urban development in formerly irrigated areas are all possible options for repurposing (52). In addition, conservation incentive programs could help mitigate the impacts of fallowing on ecosystems and people, and redistributing irrigation water onto fewer irrigated acreage should consider ecosystem services of alternative uses to maintain multifunctional landscapes in a changing climate.

### Supply-Side Practices and Policies.

Augmenting water supplies through importing water from other regions, or further tapping into local surface or groundwater supplies, are limited at best. Yet supply augmentation options do exist, albeit likely at a higher cost (51). A portfolio of options needs to be considered, including better capture and use of flood water, maintaining healthy soils, and more effective monitoring, surveillance, and response to extreme weather events. Groundwater recharge (especially during flood events), water recycling and reuse, and desalination provide opportunities to enhance supply. Increasing the operational efficiency of surface or groundwater storage and transport can also increase water availability. Last, water trading can help reallocate water supplies to reduce costs of both temporary and long-term shortfalls (30).

#### Groundwater recharge.

Managed aquifer recharge (MAR) is the intentional recharge of water to aquifers for subsequent recovery or environmental benefit (53). MAR practices have been used in California in its operation of water banks–aquifers used for underground storage–and to avoid saltwater intrusion in aquifers in coastal zones. There is now renewed interest in developing MAR efforts to catch flood flows, especially for its low financial and environmental cost compared to other alternatives (54). The California Department of Water Resources found that an annual average of almost 2,000 hm^3^ is available for recharge using current infrastructure without interfering with environmental regulations. Adding new infrastructure could increase recharge opportunities in nearly all California regions over time, and particularly in the Sacramento Valley where significant opportunities exist (55). The flows that comprise the recharge are often available in large magnitudes for short periods (e.g., extreme runoff or flooding events) and thus present challenges due to regulation and (limited) infrastructure. Current storage and conveyance infrastructure as well as operational and regulatory practices need to be expanded and improved to make full use of this water supply augmentation option.

Although most water volumes have been recharged in dedicated basins in California, there is also much interest for on-farm recharge (56). By recharging water directly on farms, current irrigation infrastructure could be used, thus reducing the costs. Institutional challenges include lack of incentives for farms to accept flows because the individual farm benefits may be small relative to the public benefits. Additionally, some crops likely are better suited for this than others, e.g., crops that are dormant in winter–such as almonds and vines–may not be negatively impacted by this practice. Additional research on recharge issues is needed to better understand the effects of on-farm recharge on crop yields, water quality, and soil health, among other factors (57).

#### Wastewater recycling (water reuse).

Treating wastewater for posterior use is another source of water for California’s farms. The California State Water Resources Control Board estimates that 900 hm^3^ of wastewater was recycled in California in 2020 (58), with 250 hm^3^ being used for agriculture. In 2020, the state published its California Water Resilience Portfolio (59), which aims to recycle and reuse 3,100 hm^3^ over the next decade. Most of the wastewater in the CV is already being used with further treatment by downstream users or the environment. Therefore, the most promising locations for wastewater reuse and recycling are in Coastal California, where much of the wastewater is not being reused. Furthermore, while wastewater quality varies significantly across sources with more highly polluted water needing more costly treatment, some of those costs might be avoided for some farm uses (60).

#### Desalination.

Salty water can be treated to make it suitable for urban or agricultural use. In California and other western states, desalination has mostly been used to remove salts from brackish water. The lower constituent concentrations in brackish water make the process less costly than ocean desalination and, thus, more feasible for farm use. Currently, 14 seawater desalination plants are spread across California producing 110 hm^3^, with another 23 brackish groundwater desalination plants producing 173 hm^3^ (61). There are plans to desalinate another 35 hm^3^ of seawater by 2030 and 104 hm^3^ of brackish water by 2040. These quantities contribute a small fraction to the overall water supply in California. Also, the infrastructure and energy costs of seawater desalination remain high (often over $2/m^3^) particularly for agriculture, even without consideration of the likewise costly mitigation of negative environmental effects. Some have identified inland nonseawater desalination as lower cost alternative (62), yet brine disposal costs at the operation scale needed for irrigation may remain a challenge. Seawater desalination is mostly used in urban areas of Southern California and the Central Coast, where alternatives are even more expensive.

#### Water trading.

California has a small active water market where buyers and sellers trade water (63). These trades–ranging from 2 to 5% of all water used by cities and farms, reduce the economic costs of shortfalls during droughts and accommodate geographic shifts in water demand, enhancing flexibility in water management (63, 64).

Studies have found that trading could bring significant benefits to agriculture, the environment, and urban users in California (27, 48, 49, 65). The benefits of an expanded water market grow as water scarcity intensifies, which is likely given the transition to sustainable groundwater use and the reduction in water availability driven by climate change (31, 65). But a combination of aging infrastructure and complex, conflicting regulatory structures, including volume limits, hinder the expansion of trading (48, 63). Improving market design, addressing impacts on third parties, securing stakeholder buy-in, and reducing transaction costs are needed to improve California’s water market (66, 67). Of course, increasing water demand by cities may further drive water from agriculture to cities through water trading agreements (67, 68).

### The Mix of Supply- and Demand-Side Options.

The combination of supply- and demand-side options will shape the evolution of California’s agriculture. With the expected water availability declines, expanding supplies could mitigate the reduction of California’s agricultural output. But economic pressures constrain supply expansion, as most supply options are too expensive for crop irrigation, which is profitable only if the revenues of the expansion outweigh the opportunity costs (27). Water trading should incentivize supply expansion, as trading allows water to move to higher profit cropping locations. Federal and state investments can also propel supply expansion.

An economic assessment of supply- and demand-side options in the SJV (51) found that around 500 hm^3^ of supply expansion (mostly through groundwater recharge) might be efficiency enhancing—i.e., willingness to pay for supplies is greater than the costs. While 500 hm^3^ only represents a quarter of the expected decline in water availability, demand reduction will comprise most of the adaptation. Other regions will have different constraints and options. In the Sacramento Valley there will be less water availability declines and more options for groundwater recharge, resulting in less demand reduction. In the Central Coast, high-value crops are more likely to pay for expensive supply options (like water recycling), but even there some demand reductions are likely. In the South Coast, growth of urban demands and the reductions in Colorado water allocations will likely be met by reduced irrigated acreage, although supply expansion partnerships between local farms and urban interests might be feasible (69).

### Cropping System Design.

For better performance, water stewardship must be accompanied by cropping system adaptations to climate change that reduce water use while regenerating natural resources, maintaining food production, and allowing farms and ranches to build resilience mechanisms. Adapting crop management practices are a main entry point for adaptation through changes in crop location, planting schedules, genotypes, and irrigation (9). The large range of crops grown in California allows for crop switching based on vulnerability assessments (70) and ecosystem service provision (71). Management complexities, response to market demand, and downstream infrastructure often make such system adjustments difficult to implement and coordinate at the watershed scale to improve water use and conservation measures.

Reallocation of water resources to perennial crops has increased in recent decades with drought-year fallowing of annual cropland. More comprehensive system-based solutions would create incentives to keep soil covered to provide cobenefits for long-term sustainability with low potential tradeoffs for water use (72). With climate change, perennial crops are increasingly exposed to year-long stressors that increase their need for irrigation and present growers with less adaptation options to annual variability, such as Relocation and replacing tree species/cultivars (73, 74). Careful implementation of low-volume irrigation systems is crucial to avoid negative implications on groundwater recharge. Moreover, while subsurface drip irrigation enhances field and plant scale water use efficiency compared to flood irrigation, drip systems can degrade soil health properties important for water infiltration and runoff control, salinity mitigation, and carbon sequestration within the soil profile (75). While efficiency and technology replacements have a role to play in optimizing water use; they seldom address the ecological, economic, and social drivers of vulnerability (76).

Effective adaptation measures must therefore be system based and consider the complex socioecological interactions at play to ensure climate smart outcomes (77). There is growing evidence that ecosystem-based adaptation options such as cropping system diversification can support adaptation while storing carbon, supporting biodiversity, and securing ecosystem services (78–81). This is especially relevant for both California’s organic crop production, and horticultural systems which tend to be more reliant on ecosystem services for pollination and biocontrol than field crops.

Managing for diversity and flexibility rather than simplification and consolidation enhances adaptive capacity by improving responsiveness to climate changes, lowering vulnerability, and allowing portfolio effects to mitigate impact of disturbances (78, 82, 83). Diversification using intercropping, longer crop rotation, or integrated crop livestock designs have been shown to support water regulation and buffering of temperature extremes as well as other ecosystem benefits which can in turn mediate yield stability and reduce risk of crop loss (84–92). Improvements in soil health associated with organic carbon inputs, soil cover, and diversification can mediate groundwater recharge and water and nutrient retention to mitigate yield loss under drought (91–95). However, tradeoffs and benefits of ecosystem-based approaches for adaptation and mitigation are context specific, and rigorous assessments of adaptive gains and water footprints are needed. As water scarcity and associated changes in crops and landscape structures unfold, developing approaches that exploit the interconnectedness of diversity at fields, operations, landscapes and food system scales with healthy ecosystems and communities will be critical for sustainable and equitable transitions.

### The Role of Institutions, Regulation, and Information.

Responding to climate change and the accompanying challenges facing agriculture in California is most effectively accomplished with inclusive and innovative approaches involving farm and rural stakeholders and policymakers using information and tools from researchers and advisors. With effective adjustments in response to climate and related water supply and demand concerns, California agriculture can become more economically, socially, and environmentally sustainable in the future. Water is central to that future.

Government water management and planning in California has long been institutionally and geographically decentralized. Many local irrigation districts and SGMA groundwater sustainability agencies develop, implement, and maintain plans to weather recurrent droughts and floods. Agencies attempt to facilitate system-wide flexibility in water allocation, which can improve resilience in the case of climate extremes. There is also a role for agencies to improve coordination among stakeholders and facilitate flexibility to allow water to flow where it contributes most to economic, environmental, and social goals. Unfortunately, these broad benefits often are not within the mandate of local agencies. Furthermore, devolution in water management to local agencies rather than to watershed-level governance, creates natural conflicts where one agency’s goals or actions may create conflict and externalities with another nearby agency given water often extends beyond any single agency’s political boundaries.

Investments in water-related data and information platforms have the potential for large payoffs by helping entities make more informed decisions. Unfortunately, despite the clear importance for practical decision making, a persistent lack of appropriate water accounting information hinders analysis of likely outcomes of water policy choices. By narrowing crucial information gaps, agencies may improve prospects for agriculture, ecosystems, and underrepresented communities as they confront less reliable and potentially lower overall, water supply allocations in the future (96). Better and timely monitoring and measurement at the watershed level will also provide a clearer picture of how actions in one part of a watershed may impact other parts of the watershed thereby providing policy makers with a more complete understanding of the consequences and trade-offs of any particular action within the watershed.

Universities and other institutions have long supported productivity growth and improved environmental performance of agriculture in California and elsewhere with research and outreach (97). R&D has contributed improved varieties, irrigation, and drainage technology and improved farm practices that have saved resources and improved environmental outcomes. Progress may come from better integrating social and biophysical sciences for socially just adaptations that value farmers’ knowledge and experience to assist in transitioning to more resilient systems. Developing a coherent research agenda to better integrate climate projections, pest/disease forecasting, soil ecosystems, new genotypes, and system designs into agricultural management is needed. More and better organized and documented open-access water data and models can make further significant contributions to informing policy and decision-making.

The high costs of water transactions, including among farmers, service areas and regions, and for groundwater recharge makes adaptation to climate change more difficult. During 2023, California facilitated some recharge efforts to take advantage of the extreme wet conditions and rebuild groundwater storage. Unfortunately, such measures fell far short of their potential. In addition to infrastructure limitations, permitting delays and other institutional constraints limited the extent of recharge. California’s adaptation to climate extremes would benefit from agile state and local regulation and coordination to facilitate recharge.

More integrated water and climate policy will follow from early coordinated and collaborative management and governance to exchange ideas and understand impacts of a wide and inclusive set of scenarios (98). Careful planning across the policy landscape could foster climate smart policies that leverage current incentives for GHG reductions and offsets to promote adaptation.

## Toward a Resilient Future for California Agriculture

Like many agricultural regions worldwide, California is facing extreme climate challenges, including increases in water scarcity and water supply variability. Growing competition for water to better support ecosystems and added regulatory oversight will continue to demand innovations to incentivize farms to produce more value with fewer resources. Innovations are often motivated by scarcity and high costs of resources, such as labor, land, and water. Moving forward, more innovation will need to be devoted to reconciling agriculture with ecosystem health, in the context of evolving knowledge and changing climate. External costs and benefits, along with third-party impacts, are likely to connect with global food market forces, to drive the direction of agricultural responses. The increased economic, ecological, and community benefits associated with enhanced knowledge of these connections will require significant efforts and commitments on the part of governments and institutions to be realized.

California can enhance climate resilience stewardship by adopting policies and practices to reduce vulnerabilities to climate extremes. Irrigation practices and technology of the recent past, such as those that ignore the importance of groundwater recharge and return flows, and adoption of permanent crops that have minimal year-to-year flexibility in water demand, are increasingly recognized as costly and risky.

California is recognizing the value of more flexible water use, both temporally in terms of reservoir storage and carryover rules and spatially in terms of water trading. Moving toward more flexible irrigation water use with smaller negative impacts on rural communities and the environment (63). Water markets can better direct water to the most valuable social uses by considering third-party impacts water reallocation.

Though climate change presents a more variable and uncertain future, it provides opportunities to adapt agricultural landscapes to better steward the environment. Bold measures are urgently needed as water availability limits have already been exceeded and adaptation pathways adequate to address these challenges require faster interventions than current trends (99). Approaches that decrease exposures to stress, reduce vulnerabilities, and enhance stress resistance and recovery, are important for California to address its climate change challenges.

California is poised to adopt ambitious measures to sustain agriculture as climate threats unfold and water scarcity increases. These measures include i) developing a capacity to integrate climate projections, pest/disease forecasting, new genotypes, and system designs into agricultural management, ii) reducing and redistributing irrigation water to recognize the value of ecosystem services, iii) improving prevention, monitoring, and surveillance of droughts and floods, and iv) leveraging GHG reduction and offset policies to promoting biodiversity, and ecosystem resilience. Effective adaptations must go beyond managing the conventionally measured impacts of water variability and toward food systems that address the market and social and ecological drivers (76). Investing in transdisciplinary research and education to support context-specific adaptations is especially relevant to address the potential social, environmental, and economic tradeoffs. Building strong and inclusive networks for research, knowledge sharing, and planning is critical to reduce mistakes and scale up the most effective measures that mitigate and adapt to a changing climate.

## Supplementary Material

Appendix 01 (PDF)

## Data Availability

There are no data underlying this work.
