# Supplementary material for: Cultivating climate resilience in California agriculture: Adaptations to an increasingly volatile water future
Source: Proc Natl Acad Sci U S A. 2024 Jul 29;121(32):e2310079121. doi: 10.1073/pnas.2310079121 (PMC11317594; doi:10.1073/pnas.2310079121)
Supplement: Supplementary file 1 — Appendix 01 (PDF) [file pnas.2310079121.sapp.pdf]

Supplemental Information Appendix

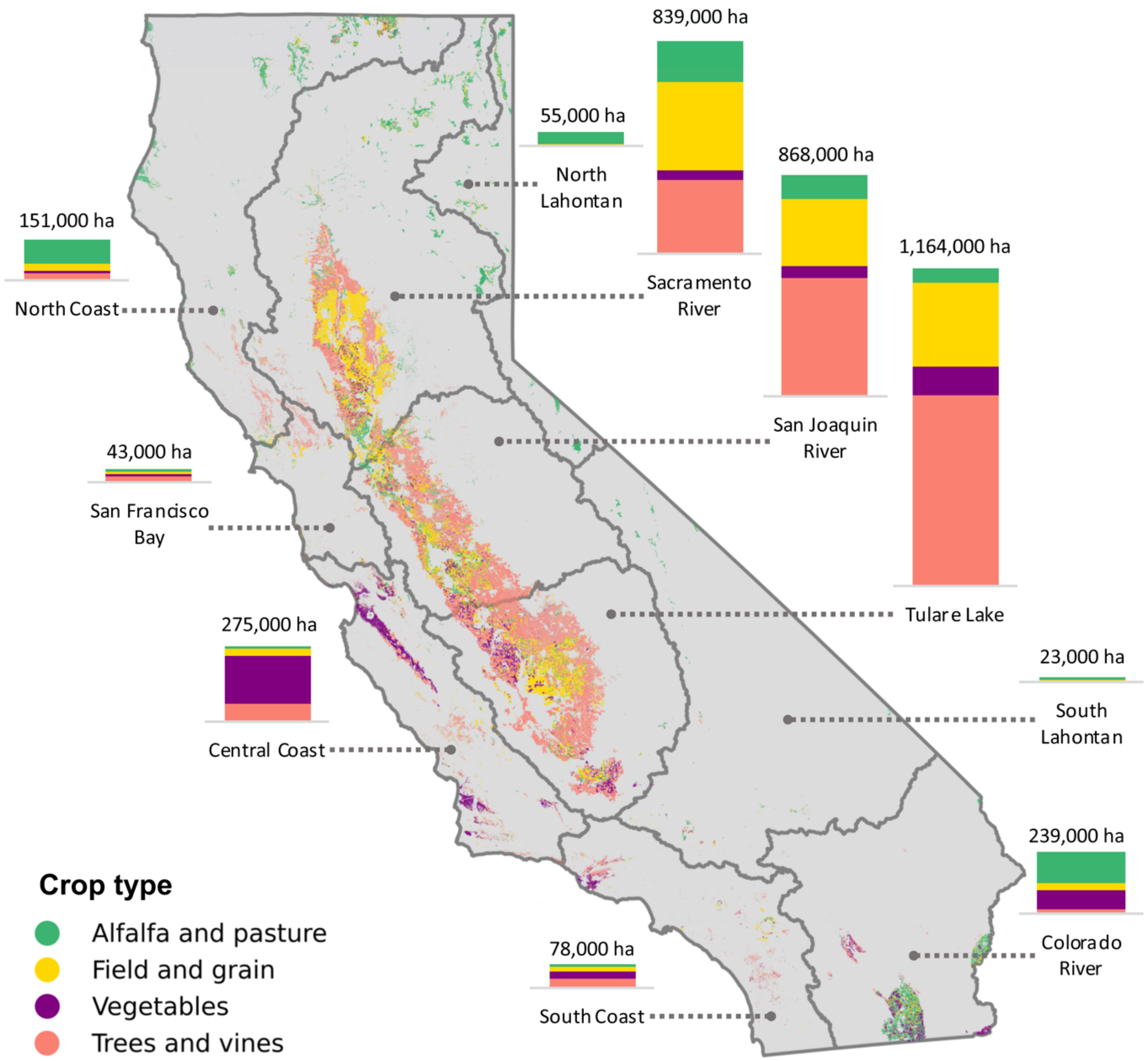

Figure S1. California Agriculture 2020. Source: California Department of Water Resources Statewide Crop Mapping 2020.
